# Biographical-Dr. P. G. C. Hunt

**Published:** 1896-08

**Authors:** H. M. Chappell, Meritt Wells, Seneca B. Brown


### Biographical—Dr. P. G. C. Hunt.

Reported before the Indiana Dental Society June 30, 1896.

The present hour has been set apart for us to pay a tribute of respect to the memory of one of our oldest members and most faithful co-laborers.

Phineas George Channing Hunt, M. D., D. D. S., who for more than half a century devoted the best energies of his life to the dental profession, has passed away.

Dr. Hunt was born in Champaign County, Ohio, in 1827, and departed this life April 24, 1896, in the sixty-ninth year of his age.

He laid down life's duties and honors in the fullness of years, surrounded by his family of loving children and a large circle of devoted friends. His wife, Hannah Mary Phillips, preceded him in 1892, leaving four children—Mrs. W. A. Crossland, Mrs. Edward Kingsbury, Dr. George E. Hunt, and Miss Luella Hunt.

Dr. Hunt's father died of smallpox in Springfield, Ohio, and the widowed mother with her children (David P., Ruth, Andrew, Phineas G. C., and Aaron) moved from Ohio and settled at West Liberty (now Knightstown) Indiana, in 1835. David and the younger brothers operated a saw-mill, which stood near where the straw-board mill now stands, and their house was across the line in Rush County.

The Doctor was a mere boy then, and his associates now living say he was a genius, and gave evidence for a bright future. The older brother (David) moved to Indianapolis and adopted dentistry, and one of his first students in dentistry before 1840 was Austin Ballard, now living in this city. Phineas G. C. went to Illinois, and then came to Indianapolis in 1845 or '46 and began as a student under his brother, and recited many of his lessons to Mr. Ballard.

In 1848 David died, and Phineas G. C. took charge of the dental practice which had been built up by the three Hunts and Ballard, and continued in business until his death. His mother and her five children all died here, and are buried near this city.

On the 28th day of September, 1858, when the Indiana State Dental Association was organized, Dr. Hunt was elected Vice-President, and was elected President in 1891, '92, '93, '94, and again in 1871.

He was a member of the temporary organization of the American Dental Association at Niagara Falls in 1859, and was present and became a member of the permanent organization of the Association in Washington, D. C., in 1860, and became its President in 1872, and he continued as a member up to the time of his decease.

In 1869 the Indiana Medical College conferred upon him the degree of Doctor of Medicine, and the Ohio Dental College conferred the degree of Doctor of Dental Surgery for his distinguished services, attainments and abilities.

As an operator Dr. Hunt ranked among the best in the world and was recognized at the head of the profession in Indiana. Several of the prominent dentists of this city and State were his students.

He was energetic, thorough, and progressive in every work with which he was associated. He contributed many valuable articles to the dental journals, and a number of his instruments, devices, and modes of manipulations, are a part of the great dental-supply trade.

Professor Joseph Richardson, in his "Mechanical Dentistry," gives Dr. Hunt credit for being the first to introduce to the profession the process of attaching porcelain teeth to metal plates by the use of rubber or celluloid, and one of the first to demonstrate the practice of replantation and transplantation to successful ends.

He was an earnest advocate, years before the practice became so popular, to insert pivot teeth on metal-pins, and to use crowns in this manner instead of extracting the roots and making plates.

Also, in various classes of crown, bridge, and porcelain-work. In fact, he was always found in advance for a higher grade of dental art and dental education.

In 1879, when we secured our first dental law, and provision was made for a Board of Examiners under the law then passed and the amended law of 1887, he was chosen President of the Board, and continued for successive re-elections until he served sixteen years, when he resigned to accept the Presidency of the Indiana Dental College.

The high standard of dental education throughout the country is greatly indebted to the efforts of Dr. Hunt, by Association work and through the Board of Examiners.

Dr. Hunt was one of the delegates from the Indiana Board to Cincinnati in 1882, when the organizatien of the National Board of Examiners was contemplated, and a temporary organization effected. The next year, 1883, he was one of the delegates to Niagara Falls, when the National Board was permanently organized. He took an active part in the business, and by reason of his being an ex-President of the National Association, which was then in session there, he manifested great influence.

During the time we were making efforts to secure the passage of our dental law, Dr. Hunt, with a number of others of us, were required to pledge ourselves to the Legislative Committee that we would orgainize a Dental College. This pledge secured the law, and when the college was organized Dr. Hunt was placed at the head of the Faculty, which place he held for years.

Dr. Hunt was a genial associate, courteous and dignified, and his personal presence was a source of inspiration, and his judgment and counsel were sought for and appreciated by those who were so fortunate as to come into a close acquaintance with him.

It is well with us that such good, gracious, and grand men as Dr. Hunt lived and shed their influence over their fellow beings. His life was a benediction, and his example was well worthy our imitation and emulation.

Dr. Hunt was born and raised a member of the Society of Friends (Quakers), and much of his simplicity of character was due to this fact.

Dr. Hunt was always kind and considerate for his juniors, and he devoted many hours of service giving instruction to the earnest seeker for knowledge and technical teaching—without hope or expectation of reward, as many of us can testify.

May we ever cherish his memory as a faithful friend and co-practitioner, and exalt his virtues; and if he possessed any faults we bury them with his remains; and may our lives be more useful as we gather inspiration from the effects of his labors.

Respectfully submitted,

H. M. Chappell,
Meritt Wells,
Seneca B. Brown.

